# Admission requirements for athletes in higher education: analysis by Pró-Atleta at the Universidade Federal de Mato Grosso do Sul, Brazil

**DOI:** 10.3389/fspor.2026.1955933

**Published:** 2026-10-21

**Authors:** Junior Vagner Pereira da Silva, Alex Caiçara de Albuquerque

**Affiliations:** Centro de Desenvolvimento de Pesquisa em Políticas de Esporte e de Lazer da Rede Cedes no Mato Grosso do Sul, Faculdade de Medicina, Mestrado e Doutorado em Saúde e Desenvolvimento na Região Centro-Oeste, Universidade Federal de Mato Grosso do Sul, Campo Grande, Mato Grosso do Sul, Brazil

**Keywords:** admissions process, athletes, higher education, public policy, Brazil

## Abstract

**Introduction:**

A career in sports sometimes requires athletes to devote a great deal of time to training and competing. This can sometimes make it difficult for them to adequately prepare for admission to higher education. Therefore, specific admissions processes or reserved spots for this group can help them prepare for life after their sports careers.

**Methods:**

This study employs a descriptive/exploratory design with a mixed qualitative-quantitative approach, based on documentary analysis and a case study. The case analyzed was the Pró-Atleta 2025 program, created by the Universidade Federal do Mato Grosso do Sul (UFMS).

**Results:**

The program's call for applications, results, and notification notices were analyzed. Fifty-three candidates applied, of whom 37 were accepted. Twenty-two candidates were admitted: 13 through general admission and nine through reserved categories. Physical Education and Physical Therapy programs accounted for 45.94% of validated applications for first-choice programs. Minimum and maximum points ranged from 50 to 4,500. Among the validated applications, state-level competitors predominated (41%), and among those selected, national competitors (36.5%) and state-level competitors (36.5%) predominated.

**Discussion:**

The UFMS Pro-Athlete program contributed to athletes' admission to higher education, particularly for state and national-level competitors.

## 1 Introduction

Reaching the elite level in sports takes five to ten years (1), with an athlete's career peak occurring between ages 20 and 30 (2). In individual sports, an athletic career lasts, on average, 17 years, beginning at age 10.7, with athletes reaching the elite level at age 14.5 and peak performance at age 23. In team sports, this process begins later and lasts longer, averaging 20.9 years: careers begin at age 10.9, elite status is reached at age 18.1, and peak performance occurs at age 24.9 (3).

The age at which elite athletes reach 18.1 coincides with holistic transitions in the athletic, psychological, psychosocial, academic, professional, and financial domains (4). Among these transitions, normative transitions stand out, characterized by predics changes such as moving from the junior to the senior category, transitioning from amateur to professional sports (5), moving from adolescence to adulthood, and moving from high school to college (4).

This series of transitions begins at age 18 (4) and brings about significant changes in athletic and academic demands (6), marking the most complex phase of an athlete's holistic development. This is the most difficult and crucial phase, as new demands related to athletic, psychological, psychosocial, academic, professional, and financial development arise simultaneously, interactively, and reciprocally, and most athletes cannot cope with them adequately (4).

Despite their dreams and investment in their athletic careers, many junior high-performance athletes have little chance of achieving these goals in college (7) and making a living from sports (8). The probability of a high-performance junior athlete advancing to the senior high-performance level is 17% among Belgian track and field athletes (9), 10% among English soccer players (10), and 10 to 18% among German soccer players (11). This challenge is even more severe for junior athletes who depend on scholarships or athletic grants to attend college. In leagues such as the *National Collegiate Athletic Association* (NCAA), only 2% of those who received scholarships in high school in the United States retained them in college (7).

Given the complexity of the transition from high school to college and from junior to senior sports, educational policies related to dual careers can help increase the number of incoming university athletes. These policies include more flexible admission requirements and specific selection processes for athletes (12). Making admission more flexible for athletes and former athletes through specific selection processes involves reserving spots for high-performance athletes.

In Europe, some Member States have formal regulations for reserving specific spots for athletes (13). Notable among these are the reservation of 3% of university spots for elite athletes and 5% for high-performance athletes in programs in physical activity and sports sciences, physical therapy, and physical education (14). In Portugal, high-performance athletes who are members of special groups are entitled to 5% of the available spots (15, 16).

Empirical evidence indicates that the adoption of reserved spots for high-performance athletes has increased in recent years, reaching 30.98% of universities in 12 European Union member states (12). Similarly, a survey of high-performance athletes at Spanish universities found that 48% of respondents were admitted to university through specific admissions processes (17).

Despite international progress, in Brazil—where a dual career model based on *laissez-faire* is in effect (18–20)—no federal legislation guarantees reserved spots for high-performance athletes in university admissions, thereby denying this benefit as a matter of state policy. However, the law grants universities academic, administrative, and managerial autonomy (21), allowing them to create specific admissions processes (22), provided they are consistent with current federal legislation. Thus, although reserving spots for athletes is not a state policy, the law allows universities to establish policies within the framework of university governance. Consequently, each institution, based on its administrators' political will, may create specific admission processes that reserve spots for athletes.

In light of the above, this study aimed to analyze the Pró-Atleta program as an institutional policy designed to facilitate the admission of high-performance athletes to the Universidade Federal de Mato Grosso do Sul (UFMS). Specifically, the study sought to analyze the relationship between the offered spots and demand for spots; identify the relationship between the available spots per program and the desired programs; investigate competition and admitted athletes by type of spot; and identify the competition levels of the athletes whose registrations have been validated and of the athletes who have been admitted.

### 1.1 Special program for the selection of high-performance athletes at the universidade federal de mato grosso do sul

In Brazil, the Indirect Administration consists of autonomous agencies, public enterprises, mixed-capital companies, and public foundations (23). Federal universities are part of this structure and are organized predominantly as autonomous agencies or public foundations (24). Education, in turn, is a social right guaranteed by the Federal Constitution and implemented as a public service provided by the State, generally carried out by entities of the Indirect Administration (25). In this context, federal universities offer undergraduate and graduate education free of charge, without charging enrollment fees or tuition, as they rely on financial transfers from the Ministry of Education.

Funded primarily by the Ministry of Education, federal universities enjoy academic, administrative, and financial and asset management autonomy (21), including the prerogative to establish admission procedures consistent with federal law, aligned with local needs, and in accordance with university leadership's aspirations.

Historically, Brazilian federal universities have used the entrance exam as the sole means of admission. Beginning in 1996, however, legislation began to allow each institution to adopt alternative selection methods (22). At UFMS, since 2010, admission has been based on the Unified Selection System and the entrance exam (26). In 2017, a new admission method was implemented: the Selective Serial Assessment Program (27).

In 2023, backed by university autonomy (21) and the legal authorization to establish specific admissions processes (22), UFMS expanded its admission options and implemented an exclusive admissions process for athletes, called the Special Selection Program for High-Performance Athletes (Pró-Atleta). Created on an experimental and temporary basis, the program aims to “[.] recognize high-performance athletes by expanding access to UFMS undergraduate programs, thereby increasing enrollment rates in higher education through the admission of candidates with recognized athletic achievements” (28, p. 255, author's translation). The first admissions cycle took place in December 2023, with the undergraduate program beginning in 2024 (28).

Selection follows a public process based exclusively on athletic merit, as shown by official documentation of results achieved in state, national, or international competitions in sports included in the Olympic, Paralympic, or Brazilian University Games. Sports achievements over the past three years are punctuated as follows: bronze medals (25 points), silver (40 points), and gold (50 points) in state competitions; bronze (75 points), silver (120 points), and gold (150 points) in national competitions; bronze (225 points), silver (360 points), and gold (450 points) in international competitions (29).

There are 103 openings distributed across 53 programs, including 37 bachelor's degree programs, 14 teacher-training programs, and two technical programs. Most programs are located at the Cidade Universitária, Campo Grande (MS), but openings are also available at the nine campuses in cities throughout the state's interior. When applying, candidates may list two programs—one as their first choice and the other as their second (29).

Despite the innovative nature of the UFMS Pró-Atleta program, little is known about its results and implications, making it important to conduct research that better explains how programs designed to make it easier for high-performance athletes to enroll in university develop. Investment in research in this area matters not only for publicizing pioneering initiatives in the country—potentially encouraging other federal universities to adopt similar policies—but also for assessing the design of implemented policies, their results, potential, and limitations. In light of political science theory, such evidence can provide important insights for institutional adjustments that improve results and broaden program reach.

Research of this nature is a valuable methodological tool, as it can identify structural anchors in national contexts, provide a frame of reference, and influence project implementation (30). Furthermore, the data generated will broaden the scope of studies on university admission models for athletes, given that the available literature originates predominantly from Europe, where federal laws—such as those in Portugal and Spain—require reserving a certain number of spots for athletes in university admissions processes.

Finally, understanding educational policies for athletes at Brazilian universities equips decision-makers with information to inform strategies for implementing programs that account for sports-education issues (31). Thus, this study helps to fill a national gap and to compare transnational models—such as the European one—with the specific characteristics of the Brazilian context through UFMS.

## 2 Materials and Methods

### 2.1 Design

This is a descriptive/exploratory study with a qualitative-quantitative approach, based on documentary analysis, and designed as a case study (32).

### 2.2 Information sources

The researchers identified the information sources and obtained the materials through formal requests to the universities via Plataforma Brasil, the official mechanism for accessing public information. The researchers consulted 69 Brazilian federal universities about policies related to dual careers, including procedures to facilitate the admission of high-performance athletes to the university.

In addition, we requested access to regulatory documents, calls for applications, and result announcements for specific athlete selection processes. Of the 69 federal universities, 66 reported that they do not have this type of program, one did not respond, and two—the Universidade Federal de Mato Grosso do Sul and the Universidade Federal de Santa Maria—reported that they have specific admission procedures for athletes. During the source identification phase, it became clear that only UFMS met the following inclusion criteria: a) the call for applications included para-sports; b) the results announcements listed the punctuation achieved by the candidates; c) the level of competition for the participants was specified.

### 2.3 Corpus and analysis techniques

The corpus consists of the following documents: Notice No. 18/2025-PROGRAD/UFMS (consolidated call for applications), Notice No. 99/2025-PROGRAD/UFMS (results of the selection process for high-performance athletes seeking admission to UFMS undergraduate programs in 2025), and Notice No. 100/2025-PROGRAD/UFMS (enrollment notice for the first round—UFMS high-performance athletes 2025).

These documents are written records that provide information for understanding facts, relationships, and contexts, making it possible to gain insight into the historical and social period in which the events took place and to reconstruct the facts and their background (32). This type of source analysis is relevant in public policy studies, as it allows structural contextualization within national contexts and international comparisons. Operationally, the content analysis technique was organized into three stages: pre-analysis; exploration of the material; and processing and interpretation of the results.

#### 2.3.1 Preliminary analysis

This stage corresponded to the preparatory phase of the analysis, before organizing the collected empirical data. An exploratory reading of the announcements was conducted, followed by the development of indicators designed to guide the understanding and interpretation of the material. The objects of analysis, the analytical indicators, and the coding procedures were defined. This initial phase represented an approach to the corpus, with a view to its subsequent operationalization.

The analysis focused on the available and filled spots in the admissions process for athletes entering undergraduate programs. The analyzed indicators (numeric registration units) were: a) the number of spots offered and the number of applications submitted; b) the ratio of spots offered to athletes admitted; c) the ratio of applications submitted to athletes admitted; d) the ratio of spots offered to athletes admitted by undergraduate program; e) the ratio of validated applications by type of athlete admitted; f) the punctuation obtained by athletes with validated applications and the punctuation obtained by those selected; g) the competition levels of applications submitted and of athletes admitted. The coding process involved calculating the absolute values of athletes' placements and scores.

#### 2.3.2 Analysis of the material

This stage involved coding, in which we identified, recorded, and organized each indicator for subsequent analysis. In the intermediate stage, we extracted the data from the documents and organized it in a spreadsheet to support subsequent analysis. We categorized the job opening data by indicator type, then analyzed absolute and relative frequencies. Specifically, for the punctuation candidates obtained, we analyzed minimum and maximum values. We organized these data into figures and a table. In addition, we calculated the competition rate (CR = number of submitted applications/number of offered spots) and the ratio of Available Positions to Approved Applications (VAR = number of approved applications/Number of available positions * 100).

#### 2.3.3 Data processing, interpretation of results, and statistical analysis

After organizing the indicators, the analytical process began by correlating the extracted figures with the characteristics of the athlete admission policy. Data analysis followed a qualitative content analysis approach: the figures were interpreted as expressions of institutional decisions, candidates' behaviors, and public policy outcomes, allowing inferences about their effectiveness, scope, and consistency. The study adopted the holistic model of sports career transition (4) and the model of categories and facilitators of dual careers (12). In accordance with National Health Council Resolution No. 510/2016, the study was not submitted to the Research Ethics Committee for review, as it is exempt from ethical review because it is a document-based analysis of publicly available information that does not contain identifiable data.

## 3 Results

The Pró-Atleta 2025 selection announcement offered 103 spots, with one spot for each program at each sectoral administrative unit, totaling 53 programs: 37 (69.81%) were bachelor's degree programs, 14 (24.41%) teacher-training programs, and 2 (3.77%) in higher education technology programs. Only medicine, law, and dentistry programs did not offer openings. Of the 103 openings, 49 (47.57%) were allocated to programs offered in Campo Grande, at the University City campus, and the remainder were distributed among the nine campuses (Figure 1).

**Figure 1:**
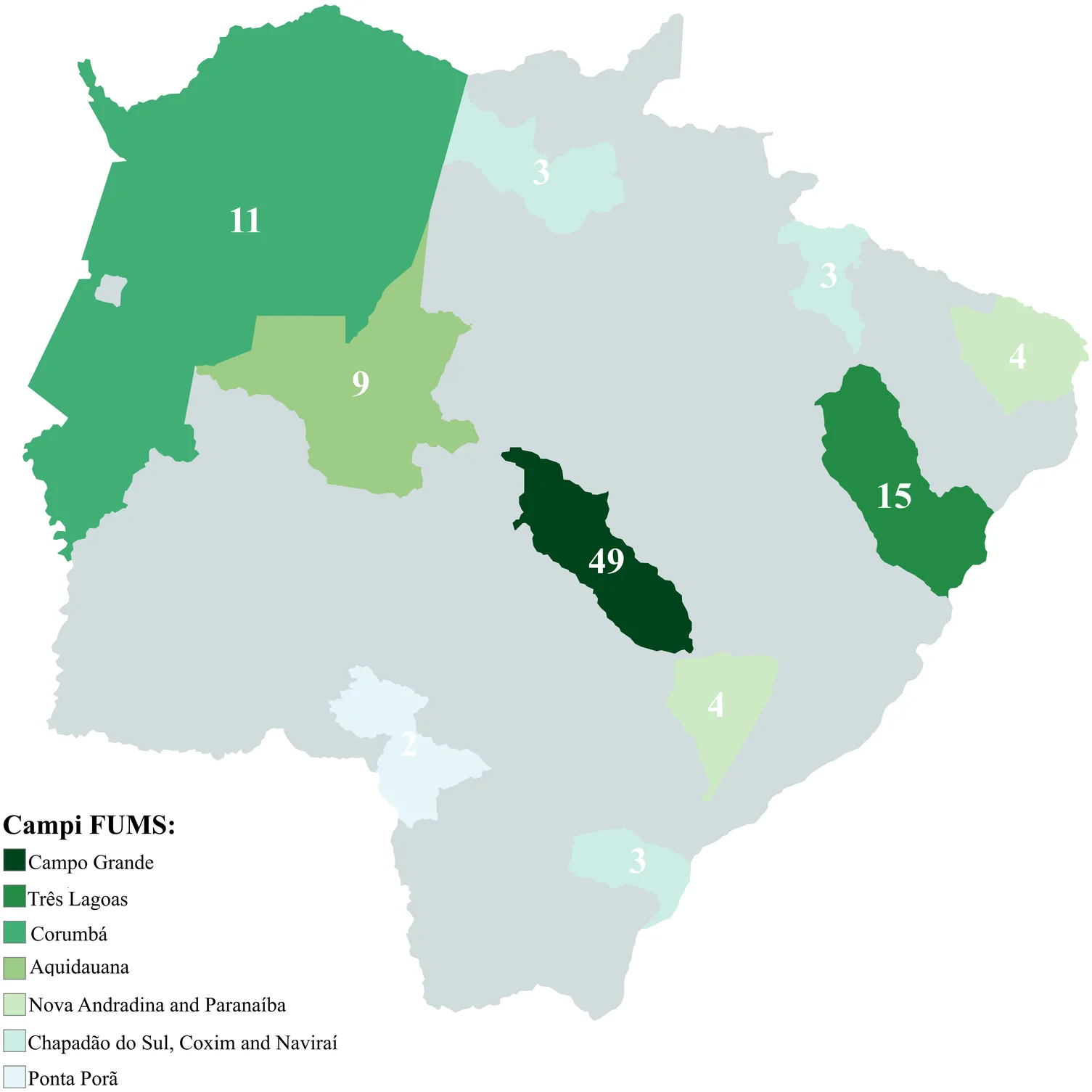
Distribution of pro-atleta 2025 spots by campus at the universidade federal de mato grosso do sul.

There were 57 applicants for the 103 openings, resulting in a competition ratio of 0.55 applicants per opening. Among the 37 applicants whose submissions were approved, 34 (91.89%) selected programs based in Campo Grande as their first choice, and 28 (75.67%) as their second choice. In the first selection option, the ratio of approved applications to available spots was 69.39% for Campo Grande and 5.56% for the Campuses. In the second selection option, these values were 57.14% and 5.56%, respectively. Additionally, six applicants did not select any program in their second selection option. Of those approved, 22 were admitted, corresponding to an admission rate of 59.46%.

As for the types of openings available under the call for applications for athlete admission to the university, 52 (50.48%) were reserved for specific groups, in accordance with the rules established by federal regulations—12 spots for people who identify as Black or Brown individuals, regardless of income (LB_PP), one spot for people who identified Indigenous people with a gross per capita household income equal to or less than 1.5 times the minimum wage (LB_I), one spot for people who identify Quilombolas with a gross per capita household income equal to or less than 1 minimum wage (LB_Q), one spot for people with disabilities whose gross per capita family income is equal to or less than 1.5 times the minimum wage (LB_PCD), ten spots for candidates with a gross per capita family income equal to or less than 1.5 times the minimum wage (LB_EP), 13 spots for people who self-identify as Black or Brown, regardless of income (LI_PP), one spot for people who self-identified indigenous people, regardless of income (LI_I), one spot for people who self-identified Quilombolas, regardless of income (LI_Q), two spots for candidates with disabilities, regardless of income (LI_PCD), and ten spots fpr other candidates, regardless of income (LI_EP). However, among candidates whose applications were validated, 22 (59.46%) competed for the general admission spots, while 15 (40.54%) competed for the reserved spots (Figure 2). The ratio of vacancies offered to those filled was higher in the general admission category (43.1%) compared to quota-based vacancies (28.8%). Among the reserved placement categories, LI_EP (100%) was the only one with full potential occupancy, while the others showed low occupancy rates: LB_EP (20%), LI_PP (15.4%), and LB_PP (8.3%); the remaining categories had zero occupancy.

**Figure 2:**
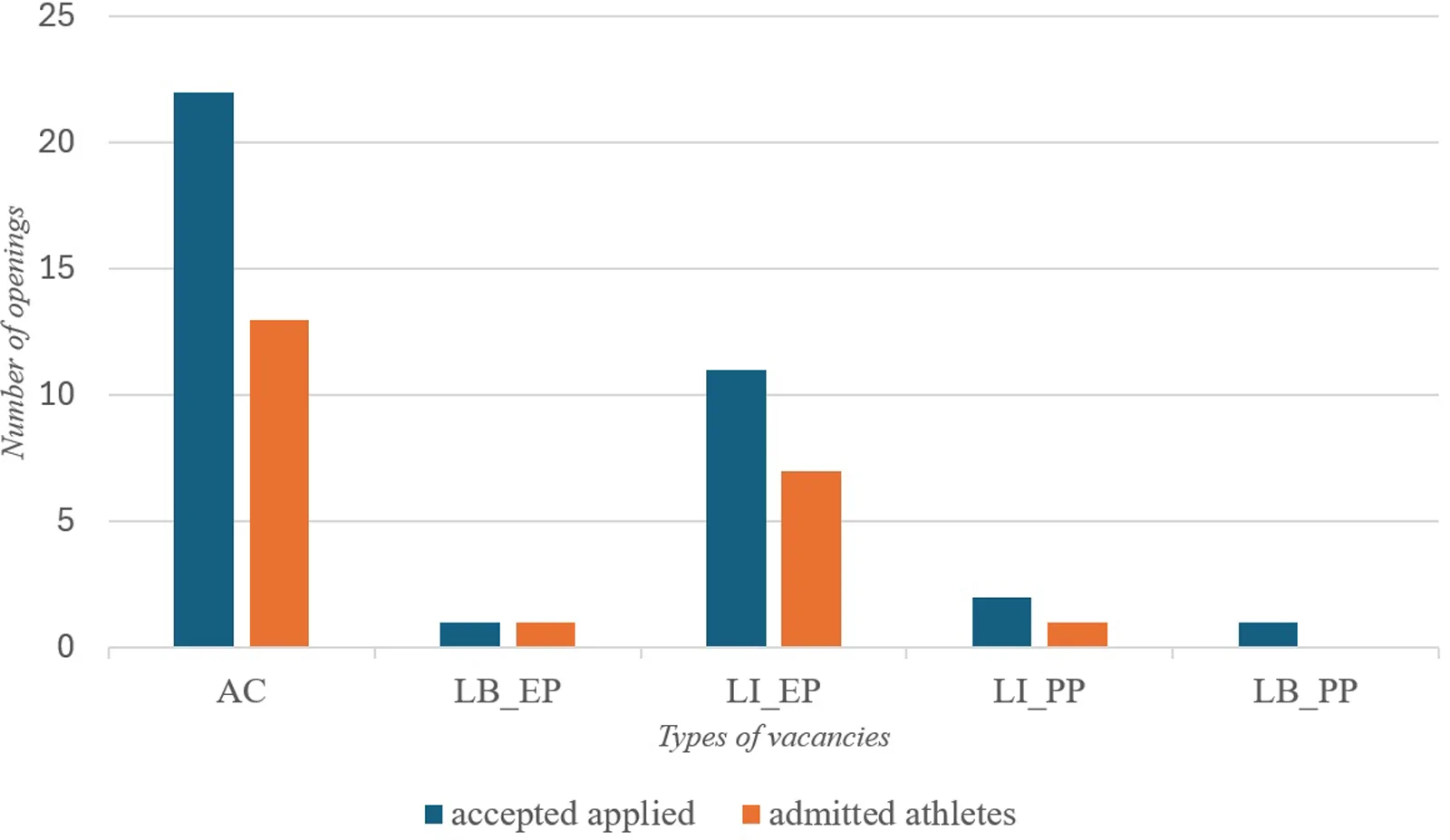
Distribution of validated applications and admitted athletes by type of spot—pro-athlete—UFMS 2025.

For their preferred programs, most athletes whose applications were validated chose physical education (24.32%) and physical therapy (21.62%) as their first choice, accounting for 45.94% of choices. For second choice, selections were evenly distributed among physical education, physical therapy, nutrition, nursing, and psychology, with 10.81% of selections for each program. Conversely, 33 (62.26%) programs– including both first and second choices–consisted of 22 (66.66%) bachelor's degree programs (agronomy, architecture and urban planning, visual arts, audiovisual studies, biological sciences, social sciences, environmental engineering, production engineering, food engineering, computer engineering, engineering physics, forestry engineering, philosophy, physics, geography, history, journalism, mathematics, management processes, information systems, tourism, and animal science), nine (27.27%) teacher-training programs (visual arts, physics, geography, humanities—Portuguese/teaching degree, humanities—Portuguese/Spanish, humanities—Portuguese/English, humanities—Portuguese/literature, music, and chemistry), and two (6.06%) higher education technology programs (management processes and chemistry—technology, as shown in Table 1).

**Table 1:** Distribution of courses requested by athletes whose applications validated applications—pró-atleta—UFMS 2025.

Undergraduate programs	Type of training	Number of openings available per course	First option		Second option	
			N	%	N	%
Physical Education	Teacher-training programs	2	2	24.32	-	-
	Bachelors's Degree	1	7		4	10.81
Physical Therapy	Bachelors's Degree	1	8	21.62	4	10.81
Nutrition	Bachelors's Degree	1	4	10.81	4	10.81
Veterinary Medicine	Bachelors's Degree	2	3	8.10	-	-
Psychology	Bachelors's Degree	3	2	5.40	4	10.81
Administration	Bachelors's Degree	9	2	5.40	2	5.40
Software Engineering	Bachelors's Degree	1	2	5.40	1	2.70
Biological Sciences	Teacher-training programs	4	2	5.40	-	-
Computer Science	Bachelors's Degree	1	1	2.70	-	-
Electrical Engineering	Bachelors's Degree	1	1	2.70	-	-
Pedagogy	Teacher-training programs	6	1	2.70	-	-
Mathematics	Teacher-training programs	6	1	2.70	-	-
Economics	Bachelors's Degree	1	1	2.70	-	-
Nursing	Bachelors's Degree	3	-	-	4	10.81
Civil Engineering	Bachelors's Degree	2	-	-	3	8.10
Pharmacy	Bachelors's Degree	1	-	-	2	5.40
History	Bachelors's Degree	5	-	-	1	2.70
Accounting	Bachelors's Degree	4	-	-	1	2.70
Chemical Engineering	Bachelors's Degree	1	-	-	1	2.70

Candidates were ranked based on points earned by providing proof of athletic achievements, ranging from a minimum of 50 points to a maximum of 4.500 points. Among the 37 candidates whose applications were validated, the most common point ranges were 100 to 199 points (16.21%), 300 to 399 points (13.51%), and 1.200 to 1.299 points (10.81%). Considering only the 22 athletes admitted in the first round, the minimum score was 50 points, and the maximum was 4.500 points. The most common point ranges among those selected were 100 to 199 points (13.63%), 300 to 399 points (13.63%), and 3.000 to 3.099 points (9.09%), as shown in Figure 3.

**Figure 3:**
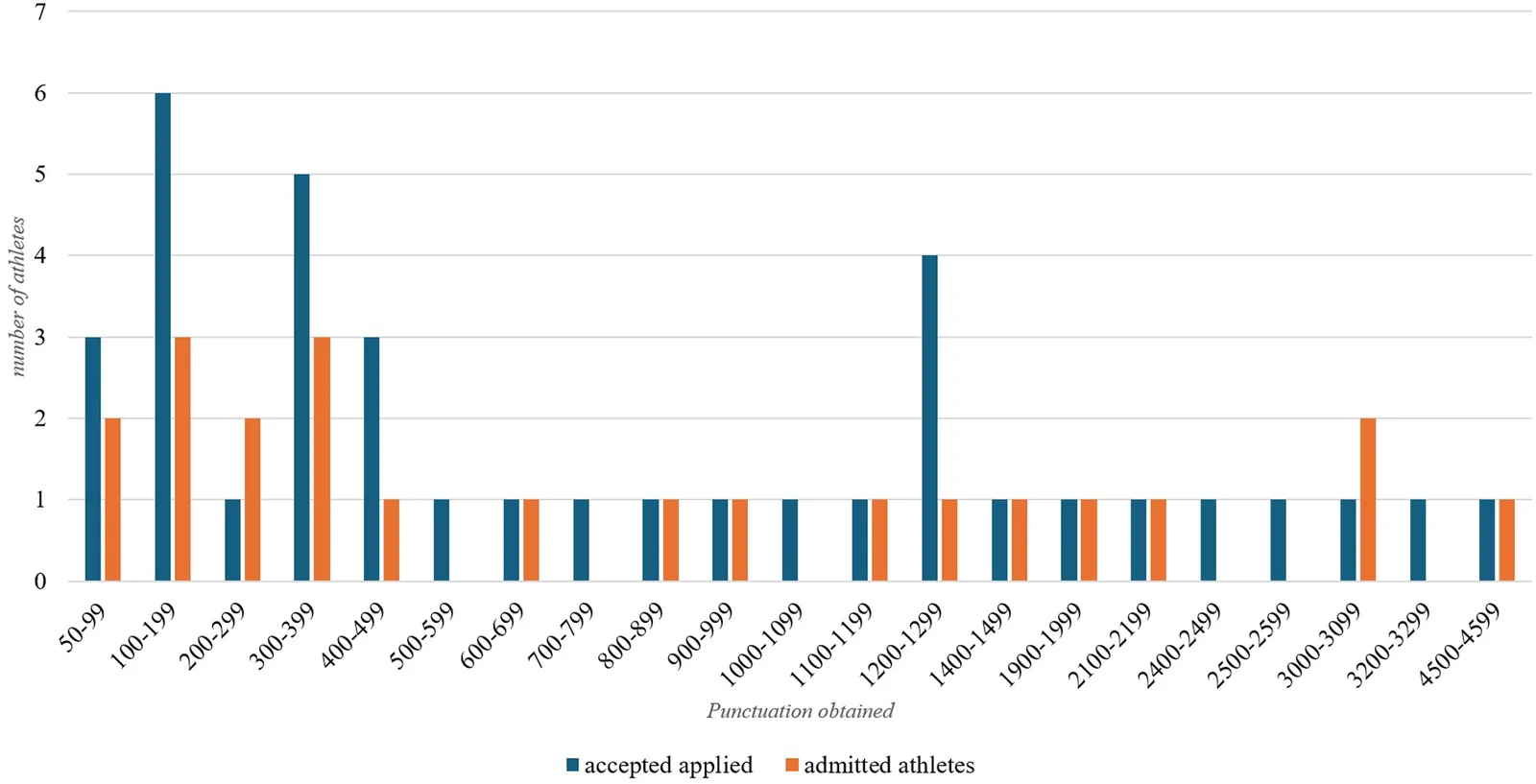
Distribution of applications obtained by validated applicants and admitted athletes under the pro-atleta—UFMS 2025 call for applications.

Regarding athletic achievements during the 2022–2024 period, among athletes with validated applications, winning a gold medal in state competitions stood out (42%) (Figure 4a). Among admitted athletes, gold medals were split evenly between national and state competitions, at 36.5% each (Figure 4b).

**Figure 4:**
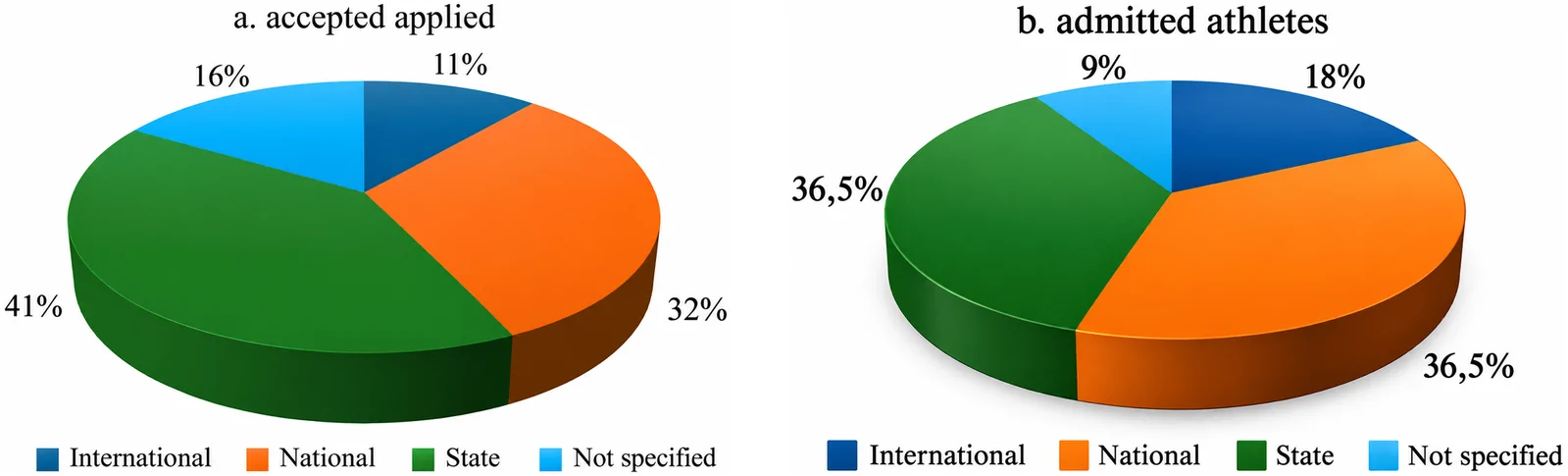
Percentage of competitive levels among validated applications and admitted athletes for the pro-atleta—UFMS 2025 program.

## 4 Discussion

### 4.1 Supply and demand for openings

The results indicate that available spots exceeded demand, with nearly three openings for every athlete who submitted an application. In addition, both the competition rate and the ratio of available positions to approved applications were low. Despite these low rates, they were higher than those observed by Silva et al. (33) in the 2024 Pró-Atleta selection process, suggesting a moderate increase in demand for spots in the program.

The low competition rate may reflect that the program is still new, meaning part of the sports community in Mato Grosso do Sul, Brazil, is not yet fully familiar with it. Conversely, the low ratio of available positions to approved applications may stem from the system for competing for spots, which begins with selecting a city and a major as first and second choices during registration. This format creates highly competitive programs, meaning athletes with strong athletic records may not be accepted because of the high level of competition in their chosen city and for certain programs. Conversely, some programs received no applicants, which also contributed to the low admission rates. This may also relate to program location, given that the ratio of approved applications to available spots in Campo Grande was 91.89% and 69.39% for the first and second selection options, respectively.

Although the competition and admission rates were low, the UFMS Pro-Atleta program is timely and highly relevant, as it expands opportunities for athletes to gain admission to the university based on their athletic performance during a complex phase of holistic development and transition. The literature indicates that athletes are highly likely to have their careers interrupted during this transition, as this phase is considered the most complex and critical stage of athletic development (5).

This transition occurs simultaneously and interactively with other normative transitions (4). It is characterized by increased competitive demands, lower physical maturity relative to opponents, and changes in coaching staff (34). In this process, new athletic, psychological, psychosocial, academic-vocational, and financial demands arise that many athletes cannot adequately address (5).

Despite the investment and the desire to pursue a career in elite sports, the chances that high school athletes who excel in sports will maintain that level of performance in college (7) or go on to become professional athletes (8) are minimal. Only a small fraction makes the transition from the junior to the elite senior level; the majority move on to recreational sports, give up sports altogether (5), or do not even enroll in college (6).

The situation becomes even more complex for athletes who depend on academic or athletic scholarships to access higher education. In South Africa, for example, there is no financial aid (35), and only 2% of students who received high school scholarships can retain them in college (7). Furthermore, scholarship benefits generally depend on maintaining athletic performance, so athletes often lose them due to declining performance or injuries (5). Thus, financial and athletic vulnerability tends to intensify precisely when athletes most need stability to balance sports and education.

In this context of complex and uncertain transitions, implementing programs to admit high-performance athletes to Brazilian federal universities—such as the UFMS Pró-Atleta program, which is based on athletic performance over the past three years—may represent an important opportunity for athletes who have dedicated a significant part of their lives to sports. However, the program did not include former athletes—those who have already retired from competitive sports and, because they no longer have performance results from the past three years, cannot access an academic education that recognizes and rewards their athletic careers by allowing them to enroll in college.

Including former elite athletes in university admissions policies is essential because low levels of education at the end of their careers are associated with professional challenges, such as a lack of specific knowledge, difficulty finding employment, and financial vulnerability. This reality was observed among former professional soccer players in Pernambuco without a college degree, who struggled to readjust to the job market after retiring from sports (36).

### 4.2 Offered spots and courses in demand among athletes

The fact that 51.45% of the validated applications were related to physical education and physical therapy highlights athletes' strong preference for these programs. Conversely, several programs received no applicants listing them as their first choice. These results are similar to those observed by Silva et al. (33) in the UFMS 2024 Pró-Atleta selection process, where physical education and physical therapy were also the most popular programs, while others received no applications.

International studies highlight strong interest in physical education and sports-related programs. Among athletes participating in the 2017 Summer Universiade, most were enrolled in sports science programs (37). At the Universidad de La Laguna, 66.70% of elite athletes were enrolled in sports-related programs (38). Similarly, among former Spanish athletes who participated in the Olympic Games, 56.3% began their post-athletic careers in sports-related professions (39).

In the specific case of physical education and sports science, this choice is often explained by athletes' strong identification with sports and physical activity, which are central elements of their daily routine and personal identity (40). This affinity serves as an intrinsic motivation—much like a love of the sport—that guides their academic choices (41). In response, some countries have adopted public policies that encourage higher education in related fields. In Spain, for example, 3% of spots in various fields are reserved for elite athletes. In contrast, in programs in physical activity and sports sciences, physical therapy, and physical education, the reserved percentage is 5% (14).

Given these factors, UFMS should evaluate increasing openings in the most sought-after programs, rather than limiting itself to one opening per program and Campus. In addition, UFMS could include programs with high social demand—such as medicine, law, and dentistry—for which it made no spots available. These measures matter because, as established in the official announcements, the Pró-Atleta spots are additional and do not reduce the number of spots programs offer through other admissions processes.

### 4.3 Competition rate and admission rate by type of spot

The regular admission pool track was the most sought-after spot type among candidates whose applications were validated, and it also had the highest number of admitted athletes in the UFMS Pró-Atleta 2025 call for applications. However, candidates with high scores (1,215, 1,080, 730, and 450) were not admitted due to the high level of competition. Conversely, 36.4% of the spots were filled by candidates in slots reserved for specific groups, as required by Laws No. 12,711 (42) and No. 14,723 (43).

Among those who validated applications for these spots, athletes with high sports résumé scores (2,475, 1,290, 575, and 465) were also not selected, further highlighting the intense competition. In Brazil, the reservation of spots for first-year university students is a legal requirement aimed at correcting historically entrenched social inequalities, such as the exclusion of Black and Brown people, Indigenous peoples, Quilombolas, people with disabilities, and those in situations of socioeconomic vulnerability who completed all of their high school education in public schools.

In the groups with reserved spots, athletes were admitted in the LI_EP, LI_PP, and LB_EP. The profile of the admitted athletes in 2025 differed from that observed in 2024. According to Silva et al. (33), that year, people who self-identified as black or brown were in the majority, regardless of income or educational background (LI_PP); people with disabilities, regardless of income, who had completed high school in public schools (LI_PcD); and students who attended public high schools, regardless of income (LI_EP). These results underscore the role of affirmative action policies in democratizing access to higher education, including in athletic admissions.

In this study, reserved spots proved important for enabling admission, particularly for athletes who completed all of high school at public schools, regardless of the reserved-spot group (LI_EP), whose seven available spots were all filled. Nevertheless, four applicants with high sports résumé scores (2,475, 1,290, 1,280, and 75) were not admitted because of intense competition.

The LI_EP group has fewer eligibility requirements, requiring only that students have completed all of their high school education at public schools. Allocating spots to this group is important for promoting social justice, given that athletic performance has historically been used to award scholarships at private schools (44).

The quota for applicants with a gross per capita family income equal to or less than 1.5 times the minimum wage, who completed all of their high school education at public or community rural schools affiliated with the government (LB_EP), allowed the only applicant registered in this category, with 385 points, to be awarded a spot. According to Wylleman and Rosier (4), the family bears the primary financial responsibility during the sports initiation phase. In contrast, during the development phase, both the family and sports organizations share that responsibility. As a result, people from socially and economically vulnerable groups have fewer opportunities for academic and athletic success, which calls for public policies that address these inequalities and promote social justice. By reserving spots for individuals in situations of socioeconomic vulnerability under the Pro-Atleta program, the university is acknowledging the adverse conditions this population faces in continuing to participate in sports and pursue higher education. Consequently, the State can promote social justice and ensure equal opportunities through government policies developed by federal universities.

Two candidates applied in the category for people who identify as Black or Brown, who completed all their secondary education at public or rural community schools, and who have a gross per capita family income equal to or less than one minimum wage. However, only one candidate, with 210 points, was accepted. In the category for individuals who identify as Black or Brown, have a gross per capita family income of one minimum wage or less, and completed high school at public schools, the sole applicant did not meet the minimum score required for admission. These results highlight the challenges associated with including Black and Brown individuals, given that applicants who are both Black or Brown and in a situation of socioeconomic vulnerability did not achieve favorable results, even in a selection process with spots reserved for this group.

Because athletes are more commonly found in private institutions, the Pró-Atleta program's policy of reserving spots in its call for applications may have expanded opportunities for these students to continue their academic education as they transition from high school to college. In the case analyzed, athletic merit was incorporated as a selection criterion, in accordance with national legislation (42, 43), which has already established this policy.

For admitted athletes, the Pro-atleta program may have provided a significant opportunity for inclusion, with dual effects, since it not only integrates this group into the university through athletic performance but also includes them in high-performance sports, thereby enabling them to compete in collegiate competitions—such as state, national, and international collegiate games—which helps sustain and extend their athletic careers.

### 4.4 Sports profile

The athletic profiles of the candidates whose applications were approved show a predominance of athletes who have competed at the state level. Among those selected, there is a balance between those with state-level and national-level experience. The profiles of dual-career athletes are diverse. College sports can include amateur, semiprofessional, and professional athletes (45). Pró-atleta athletes showed higher competitiveness than those observed at the 2018 Santa Catarina University Games, where local competitors predominated (44.2%), followed by national competitors (36.8%) and regional competitors (19%) (46). As observed in the present study and in other research, international athletes tend to be underrepresented in dual-career settings in Switzerland (47), Italy, Latvia, Portugal, Romania, and Spain (48), as well as Spain (17). Therefore, international athletes are underrepresented in dual-career contexts, as observed in Pró-atleta.

The low representation of international athletes may relate to the adoption of linear career paths (3), which are more common among professional than amateur athletes (49). In this regard, educational policies that expand athletes' access to federal universities, such as the Pró-Atleta program, could strengthen national sports and provide better conditions for the transition to life after their athletic careers, especially for athletes with diverse competitive profiles. In this context, the program can serve as an important policy for supporting athletes during their transition from high school to university, covering a range of profiles: athletes who are already at the international elite level; athletes in the process of transitioning to the international elite level; high-performance athletes in the junior category, who face a disruption in their athletic careers and the transition to the senior category; and high-performance athletes at the state level.

For athletes who have successfully transitioned from national and international elite junior-level sports—both nationally and internationally—to the senior level, being admitted to university based on their athletic achievements can represent not only recognition of their dedication and the adverse conditions imposed by their dual career, but also a reward and an opportunity to prepare for life after their athletic career. This opportunity ensures that they receive a professional education alongside their athletic training, allowing them to pursue careers in specific fields when athletic performance is no longer feasible or when they retire.

For elite junior athletes—both national and international—who have not yet reached the elite level in the senior category, being admitted to university based on their athletic performance over the past three years represents—in addition to recognition for their dedication to the sport and for the adverse conditions imposed by juggling both careers during elementary and high school—an opportunity to continue their athletic development toward the senior elite level, while also preparing academically for life after retiring from sports. It may also be a chance to join the ranks of athletes competing at the elite level in Brazilian college sports and to expand the variety of competitions available on the annual calendar, thereby promoting, to some extent, a more frequent competition schedule.

For elite junior athletes who compete at the national and international levels but are no longer competing at the highest level or are not interested in advancing to the senior elite categories, admission to college through the Pró-Atleta program can provide an immediate opportunity to transition from their athletic career to retirement, while preparing for professional life as a non-athlete. This option avoids an abrupt break from sports, as athletes in this situation can continue training and competing at the collegiate level, where the competition schedule is shorter and requires less athletic dedication compared to national and international elite competitions.

This scenario is comprehensive and consistent with the diversity of athlete profiles found in the context of dual sports careers in Brazil. It is also consistent with Brazil's characteristics as a continental country with diverse income distribution, literacy rates, sports participation, and club membership. Although dual careers are often associated with athletes who compete in major international competitions, such as world championships and the Olympic Games—that is, elite athletes (5)—some countries have adopted a broader conception in recent decades. The expanded concept of dual careers encompasses elite and semiprofessional athletes, participants in regional competitions, and third-division athletes with a high training load (50).

Given that time devoted to sport is the main complicating factor for those balancing studies and training, Bastianon and Greco (51) argue that university policies for dual careers should use time devoted, rather than performance achieved, as the criterion. This is because the challenge of balancing education and sport is the same for those who qualify for the Olympic Games as for those who, by a narrow margin, do not meet the required standards. Therefore, university admission policies that recognize the different levels of high-performance athletes—such as those highlighted in the UFMS Pró-Atleta program—demonstrate alignment with the reality not only of athletes from Mato Grosso do Sul but of Brazilian athletes in general, who, in most states, lack the club infrastructure needed to continue their athletic careers.

Specific admission policies for athletes at public universities can offer greater security and guarantees regarding the transition to life after a sports career. By being admitted based on athletic merit, athletes are guaranteed the right to an academic education, regardless of permanent injuries, declining performance, or the end of their sports career. This is because, within the Brazilian Federal Public Administration, once students pass the admissions process and enroll, they have the right to remain enrolled until they complete their degree and cannot be dismissed for poor athletic performance. This differs from scholarship programs in European countries, the United States, and private institutions in Brazil.

However, establishing admissions processes for athletes to enter university is not enough. Athletes need a support system as they begin their dual careers so they are fully prepared to succeed athletically, academically, and personally. According to European literature on dual-career guidelines for universities (12, 52), this support includes measures related to counseling and guidance, academic requirements, financial aid, social support, and policies.

The UFMS Pró-Atleta (53) program is a pioneering initiative in Brazil that allows high-performance athletes to enroll in a Brazilian federal public university through a selection process based exclusively on athletic merit. Although demand still falls short of the number of offered spots, the program has the capacity to expand access for athletes—especially those competing at the state and national levels—to undergraduate programs, helping to mitigate the challenges inherent in the simultaneous transition from high school to college and from junior to senior sports categories.

Regarding spot distribution, program choices, and the diversity of candidates' competitive levels, we observed both strengths and limitations. Key strengths include the opportunity to enroll in different academic fields, reserved spots for historically excluded groups, and recognition at the state, national, and international levels. Regarding the limitations, the following stand out: low competition in several programs, the concentration of applications in just a few fields, the absence of applicants in nearly half of the undergraduate programs, low demand for spots on Campuses, and the exclusion of former athletes.

For future editions of the Pró-Atleta program's calls for applications, it is suggested that the relevant authorities consider the possibility of: (a) expanding outreach to federations, clubs, and schools; (b) reviewing the allocation of spots in the most in-demand courses; (c) refining the selection criteria to reduce inequalities among programs and cities; and (d) including mechanisms that accommodate former athletes, recognizing their athletic careers and contributing to their educational and professional reintegration.

Overall, the program represents a significant step forward in supporting athletes, as it is the first to offer admission to undergraduate programs at federal universities based on athletic performance. Its structure may spark interest in replicating it at other federal universities, with additional adjustments to enhance equity, reach, and long-term impact, and to adapt it to each university's specific circumstances.

Although this study contributes to the body of knowledge on educational policies designed to facilitate athletes’ admission to college, its reliance on documentary data and a single academic cycle, its restriction to a single university, and the absence of athletes' and administrators' perspectives limit an in-depth analysis of the observed phenomenon. Therefore, future research should include not only UFMS but also the Universidade Federal de Santa Maria, since these are the only Brazilian federal universities with a specific admission program for athletes. Additionally, future studies should include the program's 2024, 2025, and 2026 admission cycles and, beyond document-based analyses, involve athletes and sports administrators from the institution to understand their perspectives. In addition, future studies examining the academic and athletic performance of athletes admitted through this program may also provide relevant information.

## Data Availability

The original contributions presented in the study are included in the article/Supplementary Material, further inquiries can be directed to the corresponding author.
